# Contextualising Physical Activity Levels in Patients With Anorexia Nervosa: A Systematic Review and Meta‐Analysis

**DOI:** 10.1002/erv.3205

**Published:** 2025-05-24

**Authors:** Álex V. Pagador, Hugo Olmedillas, Danika A. Quesnel, Iván Cavero‐Redondo, María Fernandez‐del‐Valle

**Affiliations:** ^1^ Department of Functional Biology University of Oviedo Oviedo Spain; ^2^ Department of Clinical Psychological Sciences University of Toronto Scarborough Toronto Canada; ^3^ Faculty of Kinesiology and Physical Education University of Toronto Toronto Canada; ^4^ CarVasCare Research Group, Facultad de Enfermería de Cuenca Universidad de Castilla‐La Mancha Cuenca Spain; ^5^ Facultad de Ciencias de la Salud Universidad Autónoma de Chile Talca Chile; ^6^ Health Institute of the Principality of Asturias (ISPA), Avda. Hospital Universitario s/n Oviedo Spain

**Keywords:** adults, anorexia nervosa, physical activity levels, teenagers, treatment stage

## Abstract

**Background and objective:**

Anorexia nervosa (AN) is a mental disorder most prevalent among adolescent females, with rising cases in younger and more culturally diverse populations. Unhealthy activity patterns are common and have been linked to increased relapse rates; however, data on objectively measured physical activity levels (PALs) in this population is scarce. This study aimed to examine PALs and sedentary time (ST) in patients with AN.

**Methods:**

Following PRISMA guidelines, 16 studies met inclusion criteria (PICO strategy). Sensitivity and subgroup analyses by age and treatment phase were performed.

**Results:**

Patients' mean age ranged from 12.6 to 36.2 years. Pooled mean ST was high (617.49 min/day). Light physical activity (LPA), moderate physical activity (MPA) and moderate‐to‐vigorous physical activity (MVPA) were higher in adult studies, while vigorous physical activity (VPA) was higher in adolescents. Steps were reported only in adult samples.

**Conclusion:**

This is the first systematic review and meta‐analysis assessing objectively measured PALs in AN patients. Findings reveal different PALs patterns by age‐group, and insufficient data by treatment stage. These results identified a critical research gap essential for future development of targeted interventions and informed strategies to support recovery.

**Summary**

- This is the first systematic review and meta‐analysis on objectively measured PALs in AN patients. Understanding physical activity in eating disorders supports tailored interventions to promote health and reduce disparities in anorexia nervosa patients.
- Teenagers spend more time in sedentary activities. Adults have higher levels of LPA, MPA and MVPA.
- In the treatment phase, inpatients show elevated values for ST, LPA and MVPA. Day‐hospital patients have the lowest MPA levels.

## 1 Introduction

Mental health disorders affect around 800 million people worldwide each year (Whiteford et al. 2016; Ritchie and Roser 2018). Of the mental health disorders, eating disorders (EDs) are particularly impactful as they cause serious harm to both the mind and body. Anorexia Nervosa (AN) is the ED with the second highest mortality among all mental illnesses (Hjemsæter et al. 2019; Van Eeden et al. 2021). This ED is most prevalent in females between the ages of 15 and 19 years (Berends et al. 2018) with increasing numbers in younger and more culturally diverse populations (Reba‐Harrelson et al. 2009). Much has been left to learn about AN treatment as recovery rates often do not exceed 50% in adults and 75% in children even with intensive treatments (Berends et al. 2018)and relapse rates are significant (∼50%). Together these factors contribute to the development of chronic eating pathology, with individuals waxing and waning between the EDs. Sadly, death from ED‐related health consequences (e.g., cardiac arrest) and death from suicide is common for people with AN (Moskowitz et al. 2017). Additionally, the rates and severity of this ED have worsened over the last decade, resulting in high health care and economic costs (i.e. the latest data reflect up to 2615 million dollars across 2018–2019 in the United States) (Deloitte 2020).

Predictors of relapse and recovery such as the symptomatology of the ED, comorbid diseases and symptoms in the treatment process (Hausenblas et al. 2008) or unhealthy exercise engagement (during the first 3 months after hospitalisation discharge) have been extensively investigated in AN (Berends et al. 2018; Strober et al. 1997). With prevalence rates between in 31% and 80% of patients depending on the age group and treatment stage, is often the first presenting and last remaining symptom in AN and is central to the pathology (Dalle et al. 2008; Alberti et al. 2013; Melissa et al. 2020).

Unhealthy exercise behaviour—more recently defined as maladaptive movement– encompasses a range of activities that are compulsive, rigid and driven by the need to regulate affect, often motivated by concerns about shape and weight (Melissa et al. 2020; Barker et al. 2022). These characteristics can be present during strenuous exercise, incidental or occupational physical activity (e.g., walking to the bus stop or working as a server) and hyperactivity (Melissa et al. 2020). Therefore, unhealthy exercise behaviours involve different intensity levels of activity and engagement time‐periods. There is no single agreed‐upon definition of maladaptive exercise, however, Barker et al. (2022) provide a broader definition that includes compulsive exercise, rigid exercise patterns, exercise avoidance, and lack of enjoyment. Additionally, their concept of ‘Exercise Satiation’ provides a theoretical framework for understanding and addressing problematic exercise in patients with AN. This concept emphasises the importance of achieving a satisfactory level of exercise to reduce distress and enhance adherence to therapeutic interventions (Barker et al. 2022).

There have been efforts to define the level of exercise engagement that characterises maladaptive exercise in AN. Traditionally, research has focused on measuring physical activity (PA) in terms of strenuous or vigorous exercise. However, recent findings indicate that unhealthy engagement in light physical activity (LPA) is more strongly linked to exercise addiction and higher relapse rates in individuals with AN (Strober et al. 1997; Dalle et al. 2008). In this sense, some studies have also identified LPA during hospitalisation to be detrimental for disease prognosis (Alberti et al. 2013; Grosser et al. 2020), whereas teenagers with AN after hospitalisation do not reach healthy PA recommendations for moderate‐to‐vigorous physical activity (MVPA) or engage in minimum levels of LPA (Agne et al. 2022). Therefore, a deeper knowledge of all types of PA by age groups and throughout the course of treatment (e.g., acute, day hospital and out‐patient regimes and recovered patients) may be crucial to aid patients with AN in transitioning from the hospital settings to real life (Ng et al. 2013; Beumont et al. 1994; Quesnel et al. 2018). Indeed, clinicians recognise the importance of activity for individuals with AN, and the growing interest of the American Psychiatric Association led to the incorporation of exercise management resources in the Practice Guideline for the Treatment of Patients with ED (American Psychiatric Association, 2023).

Regarding the assessment of PA, studies comparing self‐reported versus device‐reported measures have shown the latter to be most appropriate for a thorough identification of all physical activity levels (PALs) (e.g. light, moderate, vigorous) and engagement time (e.g. time in min/day, etc.). In fact, some studies show self‐reported tools (i.e. interviews or questionnaires) to underestimate the amount of LPA and MVPA performed by adult patients with AN. Failing to address this knowledge shortfall regarding PA engagement hinders medical teams ability to effectively support patients in acquiring the skills needed to maintain healthy and fulfiling movement behaviours post‐discharge (Bezzina et al. 2019; Bratland‐Sanda et al. 2010; Van Elburg et al. 2007).

Based on this, the purpose of this meta‐analysis was to present device‐reported data on PA engagement in recovered patients and patients with AN diagnosis across different treatment stages and age‐groups. The findings derived from this study could impact PA management (e.g. individualised assessment, re‐introduction of activity as part of a healthy lifestyle programs and life normalisation) in AN.

## 2 Methods

The review protocol was registered in advance in PROSPERO. The protocol was designed and reported following the Preferred Reporting Items for Systematic Reviews and Meta‐Analyses (PRISMA) Statement (Page et al. 2021) (see Supporting Information S1: Table S1). Changes to the initial protocol were made, including participants recovered from anorexia.

### 2.1 Search Strategy

A search was conducted using four publication databases (PubMed, Web of Science [WoS], Cochrane Libraries and Scopus) for studies published up to May 2024. Records were searched in each database using Boolean operators as follows: ((anorexia OR ‘anorexia nervosa’)) AND ((accelerometer OR accelerometry OR assessment OR levels)) AND ((‘exercise addiction’ OR ‘compulsive exercise’ OR ‘physical activity’ OR ‘exercise’)). The searches were limited to humans.

### 2.2 Eligibility Criteria

Inclusion criteria were selected following the PICO (Population, Intervention, Comparison, Outcome) strategy. Studies involving individuals with AN diagnosis and recovered patients, where is reported exercise or PALs assessed using electronic devices (e.g. accelerometers, or pedometers among other) and measuring activity in minutes or hours per day (min/day and hour/day, respectively), steps per day (steps/day); articles failing to meet the inclusion criteria were excluded. Only studies published in peer‐reviewed English‐language journals were considered. Protocol studies and reviews were excluded, also grey literature was not included in this meta‐analysis. The abstract was reviewed independently in a secondary review process rejecting those that did not meet the above‐mentioned criteria.

### 2.3 Study Selection

After applying the inclusion and exclusion criteria above, articles in which data were missing the full text was retrieved and carefully reviewed to verify the inclusion and exclusion criteria. Two reviewers (A.V. and M.F.) independently screened the titles and abstracts. Disagreements were reviewed again together, and a consensus was formed. A total of 16 studies were then identified as relevant.

### 2.4 Quality Assessment

Quality of the studies was assessed using the Joanna Briggs Institute (JBI) for quantitative analytical studies that evaluates aspects such as selection of participants, comparability of groups and outcome assessment (Moola et al. 2024). The results were summarised and presented using Cochrane Risk of Bias Generic tool (McGuinness and Higgins 2021). The results are expressed through a ‘low’ bias (no limitation), ‘some concerns’ or ‘high’ bias (severe limitation) and an overall judgement. A study with an overall ‘low risk of bias’ has a low risk for all domains, a study classified as ‘some concerns’ have certain limitation in at least one domain but no high risks, and a study categorised as ‘high’ have a high risk of bias in at least one domain or some concerns for multiple domains (Lizarondo et al. 2024).

### 2.5 Data Extraction

Data coding from each selected report was conducted by A.V. and M.F. Then, D.Q. double‐checked all the information. When there were inconsistencies, they were resolved by an additional expert (H.O.). As shown in Tables 1 and 2, data extracted included: first author's last name and publication year, sample characteristics (as sample size, mean age, age range and/or standard deviation and gender proportion), treatment stage, study design, PA assessment devices utilised for quantification, assessment protocols and PA variables registered, other assessment (including psychometric and physical health assessments among other), units (e.g. min/day, hours/day, steps/day, etc.), PA‐related results, outcomes related to other assessments (e.g. diagnostic tests or disorders severity trials, considering type of treatment, dose and regime). PALs were reported as numerical values (mean, standard deviation).

**TABLE 1:** Characteristics related to physical activity levels and other outcomes.

Reference	PA monitor	Protocol	Thresholds	Processing data	Daytime hours	Other assessments and outcomes
Hechler et al. 2008	Tracmor	Waist, 6 days (except water)	CPM‐based: LPA (low to moderate), ≤ 2109; MPA (moderate to high), ≤ 3962; VPA (high), ≤ 6605; vVPA (very high), > 6605	Not reported	Not reported	Demographic, BC (BMI, %BF)
Bratland‐Sanda et al. 2010	MTI 7164 Actigraph	Right hip, 7 consecutive days	CPM based: ≥ 1952 for MVPA	CSA Analyser	Not reported	Demographic and clinical history, BC (BW, BMI), diary registered PA, REI.
del‐Valle et al. 2010	Actigraph GT1 M	Waist, 7 days (Monday–Sunday). Minimum 10 h, epoch 1 min	CPM‐based. Children < 15 years old: ST, 0–499; LPA, 500–1999; VPA, 2000–2999; VPA, 3000–4499; vVPA, > 4499; children ≥ 15 years: ST, 0–99; LPA, 100–1951; MPA, 1952–5724; VPA, 5725–9497; vVPA > 9497.	Actilife software	Not reported	Demographic, BC (height, BM, BMI, %BF, TMM), skinfold thickness (triceps, abdominal, suprailiac area left side body), muscular strength (bench press, lateral row, leg‐press), functional mobility (TUG, TUDS), quality of life (SF‐36)
Carrera et al. 2012	Actiwatch model AW 4	Right ankle, 3 weekdays (except showers and swimming), epoch 1 min	CPM‐based: ST, < 200; LPA, < 1800; MVPA, ≥ 1800	Not reported	15	Demographic and clinical history, BC (BW, BMI), questionnaires (EDI‐2, STAI, CDI)
Alberti et al. 2013	Actiheart accelerometer	Left side chest, 3 days	METs‐based: MPA, 3–6; VPA, > 6	Actiheart software	17	Demographic and clinical history, BC (BW, BMI), questionnaires (PAQ, EDE)
El et al. 2013	SenseWear Armband	Triceps upper right arm, 4 days (except bathing or any wet activity)	METs‐based: LPA, < 3; MPA, 3–6; VPA 6–9; vVPA, > 9; MVPA, ≥ 3	SenseWear professional	Not reported	Demographic, BC (height, BW, BMI), questionnaires (EDE, BSI)
Hofmann et al. 2014	SenseWear Pro3 Armband	Not reported, 3 weekdays (Friday–Sunday) no restricted.	Not reported	Not reported	Not reported	Demographic and clinical history, BC (BW, BMI), blood samples
Fernández‐del‐Valle et al. 2014	Actigraph GT1 M	Waist, 7 days (Monday–Sunday). Minimum 10 h, epoch 1 min	CPM‐based. Children < 15 years old: ST, 0–499; LPA, 500–1999; VPA, 2000–2999; VPA, 3000–4499; vVPA, > 4499; children ≥ 15 years: ST, 0–99; LPA, 100–1951; MPA, 1952–5724; VPA, 5725–9497; vVPA > 9497.	Actilife software	Not reported	Demographic, BC (height, BW, BMI), muscular strength (bench press, leg‐press, lateral row), agility (TUG, TUDS), functional capacity
Andries et al. 2015	MTI Actigraph model GT1M	Hip, 7 days full time (except showers, swimming and sleep)	CPM‐based: LPA, 0–1951; MVPA, > 1952	Propero software	Not reported	Clinical assessments, biomarkers (hormones, IGF, urinary cortisol, adipokines), questionnaire: EDI‐2, EI (freedson combination method)
Sauchelli et al. 2015	Actical	PA (MVPA): Ankle, wrist and hip, 6 days (4 weekdays 1 weekend), epoch 1 min.	CPM‐based: MVPA ≥ 848	Actiwatch 7 software	14	Demographic, BC (height, weight, BMI, %BF), questionnaires (GHQ‐28, EDI‐2, SCL‐90‐R)
Hofmann et al. 2017	SenseWear Pro3 Armband	Dominant upper arm, 3 days (Friday–Sunday), no restrictions.	MET‐based: VPA, > 5METs	SenseWear professional	20.5	Demographic, socioeconomic, BC (BMI, BW, FM), BIA, questionnaires (PHQ‐9, PSQ, EDI‐2), blood sample
Lehmann et al. 2018	SenseWear Pro3 Armband/SenseWear Armband	Upper dominant arm, 3 days (Friday–Sunday)	METs‐based: ST 1.1–1.8; LPA, 1.8–3; MPA, 3–6; VPA, 6–9; vVPA, > 9	SenseWear software version 8.0	Not reported	Demographic, BC (height, BW, BMI), BIA
Grosser et al. 2020	SenseWear PRO3 armband	3 days (24 ± 13 days after admission adolescents; 4 ± 3 days for adults)	METs‐based: ST, 1.1 to 1.8; LPA, 1.9 to 3; MPA, 3.1 to 5.9; VPA, > 6	SenseWear professional version 8.1	17	Demographics, clinical history, sleep, BIA, questionnaires (EDE‐Q, CET, EDS‐D, OCI‐R, SCL‐27)
Langlet et al., 2021	Axivity AX3 accelerometer	Non‐dominant wrist, 24 h/day (except showers), 7 days, sampling frequency of 100 Hz	ENMO‐based: ST, ≤ 39; LPA, 40–99; MPA, 100–399; VPA ≥ 400	GGIR (R‐software)	17.5	Demographics and clinical history, sleep.
Agne et al. 2022	Actigraph 7164 MTI	Hip worn, 10 days, > 8 h/day (except showers), sampling frequency 60 Hz	CPM‐based. Children < 15 years old: ST, 0–499; LPA, 500–1999; VPA, 2000–2999; VPA, 3000–4499; vVPA, > 4499; children ≥ 15 years: ST, 0–99; LPA, 100–1951; MPA, 1952–5724; VPA, 5725–9497; vVPA > 9497.	Actilife software	10	CR fitness: TUG‐3, TUG‐10 m, TUDS, BC (BW, BMI, %BF), diet, quality of life (SF‐36)

Abbreviations: ANSOCQ, Anorexia Nervosa Stages of Change Questionnaire; BC, body composition; BF, body fat; BMI, body mass index; BSI, Brief Symptom Inventory; BW, body weight; CDI, Children´s Depression Inventory; CET, Compulsive Exercise Test; CL‐27, Symptom Checklist 27; CPM, counts‐per‐minute; EDE, Eating Disorder Examination; EDI‐2, Eating Disorder Inventory‐2; EPSQ, Exercise Participation Screening Questionnaire; GAD, Generalised‐Anxiety‐Disorder; GHQ‐28, General Health Questionnaire 28; HADS, Hospital and Anxiety Depressive Scale; K10, Kessler‐10 Item distress scale; LPA, light physical activity; MPA, moderate physical activity; MVPA, moderate‐to‐vigorous physical activity; OCI‐R, Obsessive‐Compulsive Inventory; PAQ, International Activity Questionnaire; PHQ, Patient Health Questionnaire; PHQ, Patient Health Questionnaire; PSQ, Perceived Stress Questionnaire; PSQ, Perceived Stress Questionnaire; REI, Reasons for Exercise Inventory; SCL‐90‐R, Symptom Checklist‐revised; SF‐36,Short‐Form‐36 items; ST, sedentary time; STAI, State‐Trait Anxiety Inventory; TUDS, Time Up and Down Stairs; TUG, Time Up and Go; VPA, vigorous physical activity; vVPA, very vigorous physical activity; YBS, Yale Brown Scale.

**TABLE 2:** Characteristics of the participants’ recruited in the studies included in the meta‐analysis.

Reference	City (country)	Study design	Population	Treatment stage	Sample	Sex	Age	Race/ethnicity	Socioeconomic status	BMI (kg/m^2^)
Hechler et al. 2008	Sidney (Australia)	Prospective case‐control study	Adult	OUT + DH	10 females with AN	Female	23 ± 5	Not reported	Not reported	19 ± 2.6–19.7 ± 2.5
Bratland‐Sanda et al. 2010	Vikersund (Norway)	Cross‐sectional descriptive and correlational study	Adult	IN	4 females with AN	Female	27.6 ± 9.9	Not reported	Not reported	15.6 ± 1.4
del‐Valle et al., 2010	Madrid (Spain)	RCT crossover study	Teenager	DH	22 females and males with AN (11 without resistance training and 11 in resistance training group)	Female and male	14.2 ± 1.2	Not reported	Not reported	18.2 ± 1.5–18.7 ± 1.7
Carrera et al. 2012	Netherland	Cross‐sectional descriptive and correlational study	Teenager	OUT + IN	37 females with AN (15 in the ‘warm group’ and 22 in the ‘cold group’)	Female	14.7 ± 1–15.67 ± 1.04	Not reported	Not reported	15.75 ± 1.65–16.38 ± 1.25
Alberti et al. 2013	Verona (Italy)	Cross‐sectional descriptive study	Adult	IN	52 females with AN	Female	24.4 ± 8.4	Not reported	Not reported	14.3 ± 1.7
El et al. 2013	Verona (Italy)	Prospective case‐control study	Adult	IN	53 females with AN	Female	24.5 ± 8.8	Not reported	Not reported	14.5 ± 1.6
Hofmann et al. 2014	Berlin (Germany)	Cross‐sectional descriptive and correlational study	Adult	IN	39 females with AN (10 purging type, 20 restrictive type and 9 with atypical AN)	Female	27.8 ± 2.4–28.6 ± 3.1	Not reported	Not reported	14.01 ± 0.7–14.3 ± 0.4
Fernández‐del‐Valle et al. 2014	Madrid (Spain)	RCT crossover study	Teenager	DH	36 females with AN (18 without resistance training and 18 in resistance training group)	Female	12.61 ± 0.59–13 ± 0.6	Not reported	Not reported	17.18 ± 2.55–18.12 ± 2.11
Andries et al. 2015	Odense (Denmark)	Double blind, RCT and cross‐over study	Adult	OUT + IN/OUT/IN	48 patients with AN (24 OUT + IN, 9 OUT and 15 IN)	Female	31.6 ± 10.9–36.2 ± 15.5	Not reported	Not reported	15.3 ± 2–16.4 ± 0.9
Sauchelli et al. 2015	Barcelona (Spain)	Cross‐sectional descriptive and correlational study	Adult	DH	88 females with AN	Female	27.94 ± 8.98	Not reported	Not reported	16.6 ± 1.34
Hofmann et al. 2017	Berlin (Germany)	Cross‐sectional descriptive and correlational study	Adult	IN	38 females with AN	Female	27.5 ± 8.9	Not reported	Not reported	14.8 ± 1.7
Lehmann et al. 2018	Berlin (Germany)	Prospective case‐control study	Adult	IN	50 females with AN	Female	25 ± 2.25	Not reported	Not reported	14.4 ± 2
Grosser et al. 2020	Berlin (Germany)	Cross‐sectional descriptive and correlational study	Teenager/not reported	IN	60 patients with AN	Female	16 ± NR [13–38]	Not reported	Not reported	15.4 ± 2
Langlet et al. 2021	Stockholm (Sweden)	Cross‐sectional descriptive and correlational study	Teenager	IN	54 females and 4 males with AN	Female and male	17.8 ± 6.9	Not reported	Not reported	15.5 ± 1.0
Agne et al. 2022	Madrid (Spain)	Cross‐sectional descriptive and correlational study	Teenager	DH	60 females and 3 males with AN	Female and male	13.5 ± 1.2–14.3 ± 0.	Not reported	Not reported	17.9 ± 2.1–19.7 ± 0.7

Abbreviations: AN, anorexia nervosa; DH, day‐hospital; IN, inpatients; OUT, outpatients; OUT + DH, combined outpatients and day‐hospital; OUT + IN, combined outpatients and inpatients.

### 2.6 Analysis Approach

The DerSimonian and Laird random effects method (DerSimonian et al. 2007) as used to compute pooled estimates of mean values and their respective 95% confidence intervals (95% CIs) for sedentary time (ST), LPA, moderate physical activity (MPA), vigorous physical activity (VPA) (computed as de sum of VPA and very vigorous physical activity [vVPA]), MVPA and steps (see Table 1 for classification). Heterogeneity was assessed using the I2 statistic, which ranges from 0% to 100%. According to the I2 values, heterogeneity was considered not important (0%–30%), moderate (30%–60%), substantial (60%–75%) or considerable (75%–100%; Higgins et al., 2002). The corresponding *p*‐values were also considered.

Sensitivity analysis (systematic reanalysis removing studies one at a time) was conducted to assess the robustness of the summary estimates. Subgroup analyses were performed according to age‐group; teenagers (teenagers were considered between 12 and 17 years old), adults (adults were considered over 18 years old), and the combine of adults and teenagers, and treatment phase (inpatients, day‐hospital, outpatients, recovered and combined). Finally, publication bias was assessed using Egger's regression asymmetry test (Sterne et al. 2001 jul 14). A level < 0.10 was used to determine whether publication bias might be present. Statistical analyses were performed using Stata SE software, version 15 (StataCorp).

## 3 Results

### 3.1 Selection Process and Study Characteristics

**FIGURE 1:**
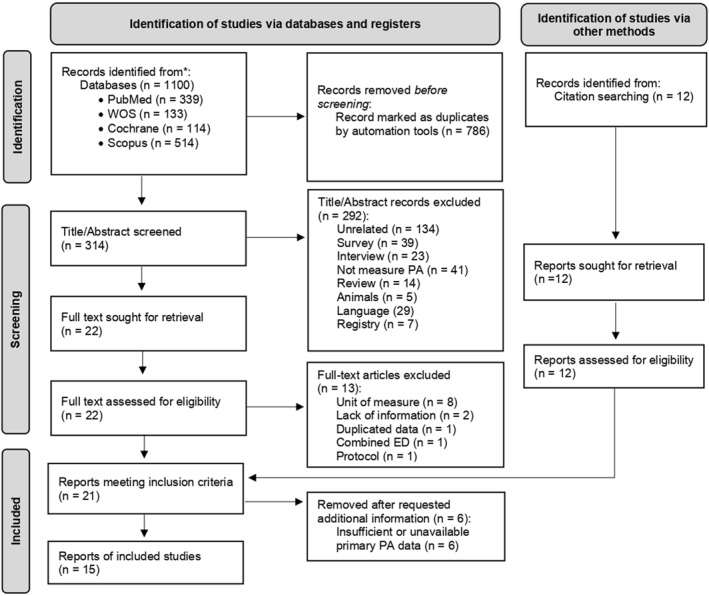
PRISMA flow diagram of the meta‐analysis.

A flow diagram of the study selection process is presented in Figure 1. The search for articles was conducted on 31 May 2024. The number of records obtained was 1100. Duplicates were eliminated in a two‐step process. First, we used Mendeley Desktop 2.79.0 software and detected a total of 773 duplicates. Then, the remaining 327 reports were transferred to the Rayyan‐Intelligent Systematic Review software (Ouzzani et al. 2016) for inclusion and exclusion assessment. Rayyan tool detected an additional 13 duplicates, leaving a total of 314 reports for screening. These were examined by reading the title and abstract, process by which 292 reports were discarded for several reasons: 134 articles were not related to AN, 39 measured PA using surveys and 23 utilised interviews, 41 did not measure PA, 14 were review articles, 5 were animal‐based studies, 29 did not use English language and seven were registries. In addition, another 12 articles were found throughout the references. The number of articles undergoing full text review was 34, of which seven articles was discarded for expressing PA in counts (defined as ‘measure that quantifies acceleration within a time interval’) (Neishabouri et al. 2022), two due to lack of information in the description of physical levels, one article was discarded due to duplicated data, one combined several ED and one study did not measure PA daily but rather at specific time‐points (e.g., at lunch time or while watching TV). A total of 22 articles met the inclusion criteria and were examined to extract the data. Following data extraction, we contacted the authors of all selected articles to request any missing or incomplete data needed for our analysis. Although we received responses from six authors, these studies were ultimately excluded due to insufficient or unavailable primary PA data. Reasons for exclusion included lack of access to original datasets or information that did not meet our criteria within the required timeframe. A total of 16 studies were included in this meta‐analysis.

Of the 16 reports included in this meta‐analysis, 15 were conducted in clinical facilities in Europe and 1 were carried out in Australia. In addition, designs were as follows: 10 cross‐sectional studies, three prospective case control‐studies and three were randomized controlled trials (RCT).

Table 2 shows the characteristics of the participants´ recruited in the studies included in the meta‐analysis. The total number of the subjects was 726 (719 females and 7 males) therefore, 99% were females. Regarding the treatment stage, 424 were in an inpatient hospital or in an intensive care unit (IN), 209 in a day‐hospital regime (DH), nine in an outpatient care (OUT), 13 identified as recovered (REC), 10 combined outpatient and day‐hospital regimes (OUT + DH) and 61 combined inpatient and outpatient regimes (IN + OUT). Patients age range was 12–65 years, and mean age varied from 12.6 to 36.2 years. Body mass index (BMI) varied from 14.01 to 25.3 kg/m^2^ depending on the study.

Table 1 shows the characteristics related to PA and other outcomes of all the studies. Briefly, cutoff points defined corresponded to ST, LPA, MPA, VPA, vVPA and MVPA values were reported using min/day (or hours/day). Steps per day were also reported. For easier interpretation, units of measure of 4 reports were transformed to min/day (Andries et al. 2015; Carrera et al. 2012; Duriez et al. 2021; El et al. 2013), and VPA and vVPA data were transformed into VPA (Alberti et al. 2013; Grosser et al. 2020; Agne et al. 2022; Bezzina et al. 2019; Duriez et al. 2021; Fernandez‐del‐Valle et al. 2014; Hofmann et al. 2014; Langlet et al. 2021; Lehmann et al. 2018; Hechler et al. 2008). Additionally, PA values were extracted from figures on one study (Duriez et al. 2021). Protocols utilised included different placement locations (e.g. waist, upper arm, wrist, ankle, and chest), and wear time required differed among studies ranging from a minimum of 3–7 days maximum. In most of the cases, the participants were instructed to remove the device when sleeping, showering or swimming.

### 3.2 Risk of Bias and Quality Assessment in Individual Studies

According to the assessment conducted using the JBI tool (Munn et al. 2014) five reports (31.25%) showed a low risk of bias, eight reports (50.00%) showed a moderate risk of bias, and three studies (18.75%) showed a high risk of bias (see Supporting Information S1: Table S2). In three studies (18.75%), the inclusion criteria were either partially described or lacking. A total of five studies (31.25%), did not accurately describe the characteristics of the sample. In seven studies (43.75%), the confounding factors such as treatment phase of the patients or sex were unclear, and the strategies to address confounding factors were lacking in eight of them (50.00%). Finally, three studies (18.75%) the statistical analysis implemented could be improved (see Supporting Information S1: Figure S1 for detailed information).

### 3.3 Meta‐Analysis

**FIGURE 2:**
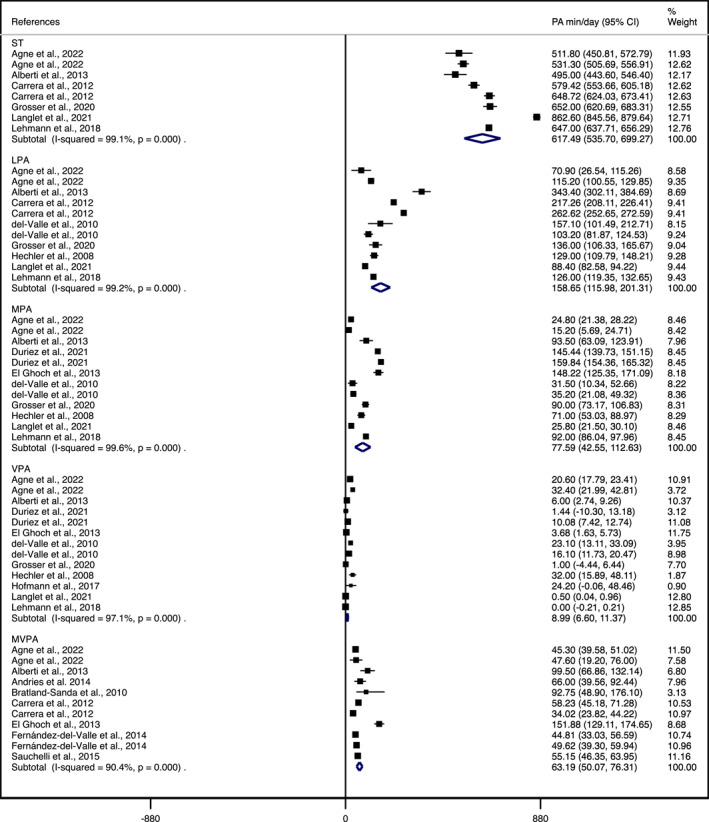
Forest plot of meta‐analyses of physical activity levels and sedentary time reported in minutes per day (min/day). LPA, light physical activity; MPA, moderate physical activity; MVPA, moderate‐to‐vigorous physical activity; PA, physical activity; ST, sedentary time; VPA, vigorous physical activity (including both vigorous‐to‐very‐vigorous PA).

Figure 2 shows the meta‐analysis of sedentary behaviour and PALs reported in min/day. The analysis revealed the following pooled mean values: average ST was 617.49 min/day (95% CI, 535.7–699.27 min/day), LPA 158.65 min/day (95% CI, 115.98–201.31 min/day), MPA was 77.59 min/day (95% CI, 42.55–112.63 min/day), VPA was 8.99 min/day (95% CI, 6.60–11.37 min/day) and MVPA was 63.19 min/day (95% CI, 50.07–76.31 min/day).

Results on additional sub‐analyses by age‐group and treatment phase are shown in Table 3 (see Supporting Information S1: Figures S2–S6, for additional information). Based on age‐group, the mean value for ST was lower in adults compared to teenagers and studies that combined both age groups. The values of LPA, MPA and MVPA were higher in adults compared to studies that included only teenagers and those that included both teenagers and adults. In the case of VPA, values were shown to be greater in teenagers compared to adults or those values reported in studies who included both populations. Lastly, daily steps' mean value was only reported for adults and studies including both teenagers and adults, leaving teenagers‐only engagement unknown. Daily steps mean value was 9761.62 steps/day (95% CI, 8444.57–11078.68 steps/day; see Supporting Information S1: Figure S7).

**TABLE 3:** Physical activity levels analysed by age‐group and treatment phase.

	Pooled estimate (95% CI)					
	ST	LPA	MPA	VPA	MVPA	Steps
Age‐group						
Teenager (12–17 y)	627.57 (480.73 to 774.41)	145.51 (79.86 to 211.17)	24.80 (20.77 to 28.84)	18.06 (5.52 to 30.60)	45.81 (39.76 to 51.86)	Not reported
Adults (18–65 y)	573.19 (424.29 to 722.08)	195.58 (118.08 to 273.08)	100.41 (74.06 to 126.77)	5.47 (1.05 to 9.89)	92.67 (49.97 to 135.38)	10.052 (8413.86 to 11691.85)
Teenager + adults	652.00 (620.39 to 683.31)	136.00 (106.33 to 165.67)	90.00 (73.17 to 106.83)	1.00 (−4.44 to 6.44)	Not reported	8.768 (6870.3 to 10665.27)
Treatment stage						
IN	665.52 (530.82 to 800.22)	165.73 (122.14 to 203.22)	89.17 (44.64 to 131.80)	1.36 (0.27 to 2.45)	119.44 (77.23 to 161.65)	9761.62 (8444.57 to 11078.68)
DH	528.28 (504.77 to 551.99)	109.02 (88.16 to 129.89)	24.64 (17.36 to 31.92)	21.31 (16.32 to 26.31)	48.03 (43.99 to 52.06)	Not reported
OUT	Not reported	Not reported	Not reported	Not reported	Not reported	Not reported
OUT + DH	614.17 (546.26 to 682.08)	203.59 (144.20 to 262.97)	71.00 (53.03 to 88.97)	32.00 (15.89 to 48.11)	45.77 (22.05 to 69.48)	Not reported
OUT + IN	Not reported	Not reported	Not reported	Not reported	66.00 (39.56 to 92.44)	Not reported

Abbreviations: DH, day‐hospital; IN, inpatients; LPA, light physical activity; MPA, moderate physical activity; MVPA, moderate‐vigorous physical activity; OUT, outpatients; OUT + DH, combined outpatients and day‐hospital; OUT + IN, combined outpatients and inpatients; ST, sedentary time; VPA, vigorous physical activity.

When analysing PALs by treatment phase, analyses revealed higher mean time spent on ST in patients under IN treatment (ST_IN_) in contrast to other treatment regimens (ST_IN_ of 665.52 min/day [95% CI, 530.82–800.22 min/day]). Likewise, patients under IN treatment and OUT + DH showed higher values time spent in LPA (LPA_IN_: 165.73 min/day [95% CI, 122.14–203.22 min/day]; LPA_OUT + DH_: 203.59 min/day [95% CI, 144.20–262.97 min/day]). Additionally, patients under DH treatment showed the lowest LPA values (LPA_DH_: 109.02 min/day [95% CI, 88.16–129.89 min/day]). Lastly, REC patients displayed the highest values on MPA (MPA_REC_: 159.84 min/day [95% CI, 154.36–165.32 min/day]), and patients under DH treatment and combined OUT + DH showed higher mean values on VPA (VPA_DH_: 21.31 min/day [95% CI, 16.32–26.31 min/day]; VPA_OUT + DH_: 32.00 min/day [95% CI, 15.89–48.21 min/day]) and IN group presented the highest mean values on MVPA with a total of 119.44 min/day (95% CI, 77.23–161.65 min/day).

### 3.4 Sensitivity Analysis and Publication Bias

The pooled mean of ST, LPA, MPA, VPA and daily steps was not significantly modified (in magnitude or direction) when data from individual studies were removed from the analysis one at a time. Finally, evidence of publication bias was observed using Egger’s test for VPA (*p* = 0.002) and MVPA (0.073).

## 4 Discussion

This is the first systematic review and meta‐analysis to examine objectively measured PALs in patients with AN across treatment phases and age. Our analyses showed elevated times spent in LPA and ST in the whole sample; however, PALs and ST profiles differed when examined by treatment phase and age‐group. These results have strong implications for the comprehensive assessment and management of PA throughout the course of treatment.

Currently the evaluation and management of maladaptive movement is non‐systematic. Clinicians seem to seldom (if at all) assess the level, type, duration, and intensity of exercise, or use guidelines for its management (Bratland‐Sanda et al. 2010; Hechler et al. 2005) Even when treatment centres do assess exercise behaviours, this process has not been supplemented by input from an exercise professional or include any type of concrete guidelines for management (Bratland‐Sanda et al. 2010). Further yet, the initial assessments may only include the question ‘are you physically active’ and vary between geographical locations without any follow‐up questioning (Bratland‐Sanda et al. 2010). In recent years, conclusions have been made about the need assessment maladaptive movement both through self‐reported questionnaire and semi structured interview (Noetel et al. 2016). Supportively, Dittmer et al. (2018) have offered suggestions for semi structured interview questions that's might be helpful. However, when it comes to PA studies have consistently shown the underreporting of PA for individuals with AN (Van Elburg et al. 2007; Dellava et al. 2011). The differences between the number of steps (*M* = ≈10.000) found in our samples compared to the intensity of the activity (ST, LPA, MPA, VPA and MVPA) presents an important, yet often unnoticed differences in the overall quantity of energy expenditure. The differences in energy expenditure can be impactful to patient's safe activity engagement and overall recovery. As such, it may be beneficial to use an objective method to assess activity intensity during treatment (Dellava et al. 2011; Dittmer et al. 2018; Meyer and Touyz 2011), with consideration to the dysfunctional use of fitness monitors in this population (Grosser et al. 2020). Hence, it may be most helpful to use ‘blinded’ activity monitors.

The patterns of PA intensity differences between phases of treatment highlight the needed considerations for LPA and MPA. As seen, VPA is the least prevalent type of activity across treatment phases and age groups. Which presents a discrepancy in the type of PA typically restricted and focused on during treatment. Rather, LPA and MPA are notably high, prior to treatment, reduce during inpatient treatment or day hospital and when individuals are discharged seem to be the main source of activity post treatment. This pattern highlights that individuals do return to movement post discharge suggesting the need for support in helping patients manage their relationship with PA over the course of treatment. Results also highlighted a difference in the MPA levels in adults and teenagers. This difference may be accounted for by the occupational and day‐to‐day demands of adulthood (i.e., grocery shopping, mowing the lawn etc.), but might be also caused by the differences in treatment received by teenagers in comparison to adults (Dalle et al. 2021). With that, treatment of youth may be more directed and supervised, meaning that youth populations are indeed restricting their movement based on treatment directives. This raise ethical concerns as we are preventing patients ‘from partaking in socially acceptable healthy lifestyle behaviours and take control of their recovery’ (Cook and Leininger 2017). In the case of adults, who presented a greater amount of LPA, MPA and MVPA, the management approach should not only focus on understanding better the nature of the movement (occupational vs. exertion). Incorporating strategies to tackle the relationship with movement in adults is of great importance, as it is inevitable to engage in such activities as treatment progresses.

There is a complex interplay between ED recovery and the incorporation of PA in ED treatment. The American Psychiatric Association’s guidelines for ED treatment recommend that PA be gradually reintroduced based on an individual’s physical and psychological health (Crone et al. 2023). Recent reviews have explored specific physical and psychological health markers that should be considered when reintroducing PA throughout treatment (Quesnel et al. 2023). One potential goal for recovery is for patients to eventually meet the World Health Organization (WHO) guidelines, which recommend at least 1 hour of MVPA daily for children and teenagers (ages 5–17). This should include vigorous‐intensity aerobic activities and muscle‐ and bone‐strengthening activities at least 3 days per week. Similarly, WHO recommends that adults (ages 18–64) engage in 150–300 min per week (∼20–45 min per day) of MPA or 75–150 min per week (∼10–20 min per day) of VPA, along with muscle‐strengthening activities involving all major muscle groups two or more days a week (Geneva: World Health Organization 2020). Since patients in the reviewed studies are at different stages of treatment and may not yet have remediated their health markers or maladaptive beliefs about activity, it's unsurprising that many do not meet these PA recommendations. For instance, teenagers in all the studies reported MVPA levels below 60 min per day (see Supporting Information S1: Figure S8 for a graphical representation) (Geneva: World Health Organization 2020; Martinez‐Gomez et al. 2010, 2011; Baumann et al. 2018; Van Dyck et al. 2015). To put this in context, this level of movement is less than that of active housework, such as cleaning a car or vacuuming, which requires about 4.3 METs (defined as ‘the amount of oxygen consumed while sitting at rest’ [3.5 mL oxygen per kilogram body weight per minute]) (Jetté et al. 1990; Herrmann et al. 2024). Longstanding ideologies recommending abstinence from all types of PA (including incidental activity, exercise, and sport) may contribute to these low PALs (Quesnel et al. 2018). Consequently, patients in treatment often experience reduced functional capacity, muscular strength, and body composition (Grosser et al. 2020; Duriez et al. 2021; Dittmer et al. 2018), which could be improved with safe, supervised and nutritionally supported PA engagement. However, many clinicians report a lack of knowledge and confidence in incorporating PA into treatment plans (Quesnel et al. 2018). This highlights the need for more clinician training on managing PA during ED treatment. Additionally, moving away from an abstinence‐based approach to PA through ‘top‐down’ programme wide ideological shifts has proven effective in advancing the field in this area (Quesnel 2022).

### 4.1 Strength and Limitations

This meta‐analysis is not without limitations. Some limitations are common to meta‐analyses (e.g., limited availability of detailed information in study reports and selection bias), however, others are particularly relevant to our study. First, the number of studies is limited as shown by the lack of data at some physical activity measures overall, by age groups or treatment stages. For example, there are no studies including only teenagers, day hospital, outpatient or recovered patients that evaluated the number of steps per day performed. Nonetheless, the major limitation relates to the availability of data by treatment stage, as most studies analysed inpatient and day hospital regimes with no data found for outpatient treatment. Moreover, this circumstance did not allow for PALs assessment by age‐group on each of the treatment stages. Second, another important limitation relates to the perception of accelerometry as a form of constraining autonomy by patients, especially those under intensive treatment regimens which may explain attrition rates in some studies. Third, the groups studied were mainly Caucasian females and do not have any information about their socio‐economic situation, therefore, our results are not generalisable to other ethnic groups or male patients.

Several methodological factors may have influenced our study results and contributed to heterogeneity. Firstly, discrepancies in the reduction of accelerometer data were evident. Specifically, the cut‐off points defining PALs varied in our meta‐analysis: LPA ranged from 500 to 1951 counts per minute, MPA from 1952 to 5724 counts per minute, VPA from 5725 to 9497 counts per minute and MVPA ≥ 1952 counts per minute. These variations could lead to significantly different estimates of time spent in each PALs. Additionally, the duration of epochs (defined as a stored magnitude of accelerations at fixed recording intervals 1, 4, 15, 60 s or longer]) plays a crucial role; different epoch lengths markedly impact accelerometer outcomes (Ayabe et al. 2013; Migueles et al. 2017). Other factors include the total number of monitoring days, criteria for non‐wear time, and the minimum daily wear time required for analysis. Another limitation of our study is the exclusive focus on step count as a measure of physical activity. While accelerometry provides an objective approximation of movement, it does not capture small, spontaneous movements known as ‘fidgets’. According to Levine (2023), these movements, although seemingly insignificant, are neurologically regulated and can significantly impact total energy expenditure. Levine emphasizes that ‘fidgets’ are rhythmic, programmed movements that substantially contribute to non‐exercise activity thermogenesis (NEAT). Therefore, by not considering these movements, we may underestimate the total energy expenditure of the patients, potentially influencing the interpretation of our results (Levine 2023). These methodological factors likely influenced our study results and contributed to the heterogeneity, as the methods for processing PA data varied widely across studies.

This study also has important strengths. The methodology applied for the review as well as the treatment of the data, allowed to identify gaps in the research that are crucial for clinicians, patients and researchers alike. In addition, this is a pioneer study, as it is the first to provide objectively data and summarized information of different descriptors of PA (e.g., ST, LPA, MPA, VPA and MVPA) in AN. Looking at a single descriptor of PA in AN—such us steps per day or vigorous intensity activity—could have led to misinterpretation of the findings. This is particularly important in AN, where maladaptive movement can manifest in different forms.

### 4.2 Conclusion

Overall, results from this systematic review and meta‐analysis revealed different PA engagement among age groups and treatment stages. Despite the differences in PALs between age groups, there is a need to improve a healthy participation in active lifestyle. However, it is also important to acknowledge the regular patterns arising from our meta‐analysis, so future efforts are directed to attend to those that are problematic. This evidence should drive the change in the maladaptive movement evaluation approach including the quantitative dimension—not only qualitative dimension—of movement. Then, medical professionals would have the necessary tools to tackle PA management during AN treatment through individualised recommendations; further, permitting patients to participate in their recovery process by learning the skills needed to engage in healthy exercise within the context of treatment before they are discharged.

Future efforts should examine PALs at multiple treatment stages, with a wider variety of patients (e.g., age, sex, ethnic groups, treatment regime among other) and larger sample sizes. Standardising methodologies for PA is essential. Using identical accelerometer devices, consistent placement sites –such as the waist, which some studies recommend as an optimal location—and standardized monitoring durations—7‐day measurement period may provide a reliable representation—would be crucial to unify PA measurement criteria. Additionally, conducting profiling studies (e.g., daily schedules) and assessing barriers to engaging in healthy PA could help identify other key determining factors. These efforts could allow researchers to design patient‐specific interventions, improve healthy active lifestyle engagement, and reduce maladaptive movement‐associated health disparities of patients with AN.

## Ethics Statement

In this review and meta‐analysis, all the information was gathered from previously published studies. The review protocol was registered in advance in PROSPERO (International Prospective Register of Systematic Reviews, no. CRD42022368432).

## Conflicts of Interest

The authors declare no conflicts of interest.

## Supporting information

Supporting Information S1

## Data Availability

The data that support the findings of this study are available on request from the corresponding author, (M.F.V.).
