# Supplementary figures and images for: Contextualising Physical Activity Levels in Patients With Anorexia Nervosa: A Systematic Review and Meta‐Analysis
Source: Eur Eat Disord Rev. 2025 May 24;33(6):1204–18. doi: 10.1002/erv.3205 (PMC12547377; doi:10.1002/erv.3205)

## Slide 1
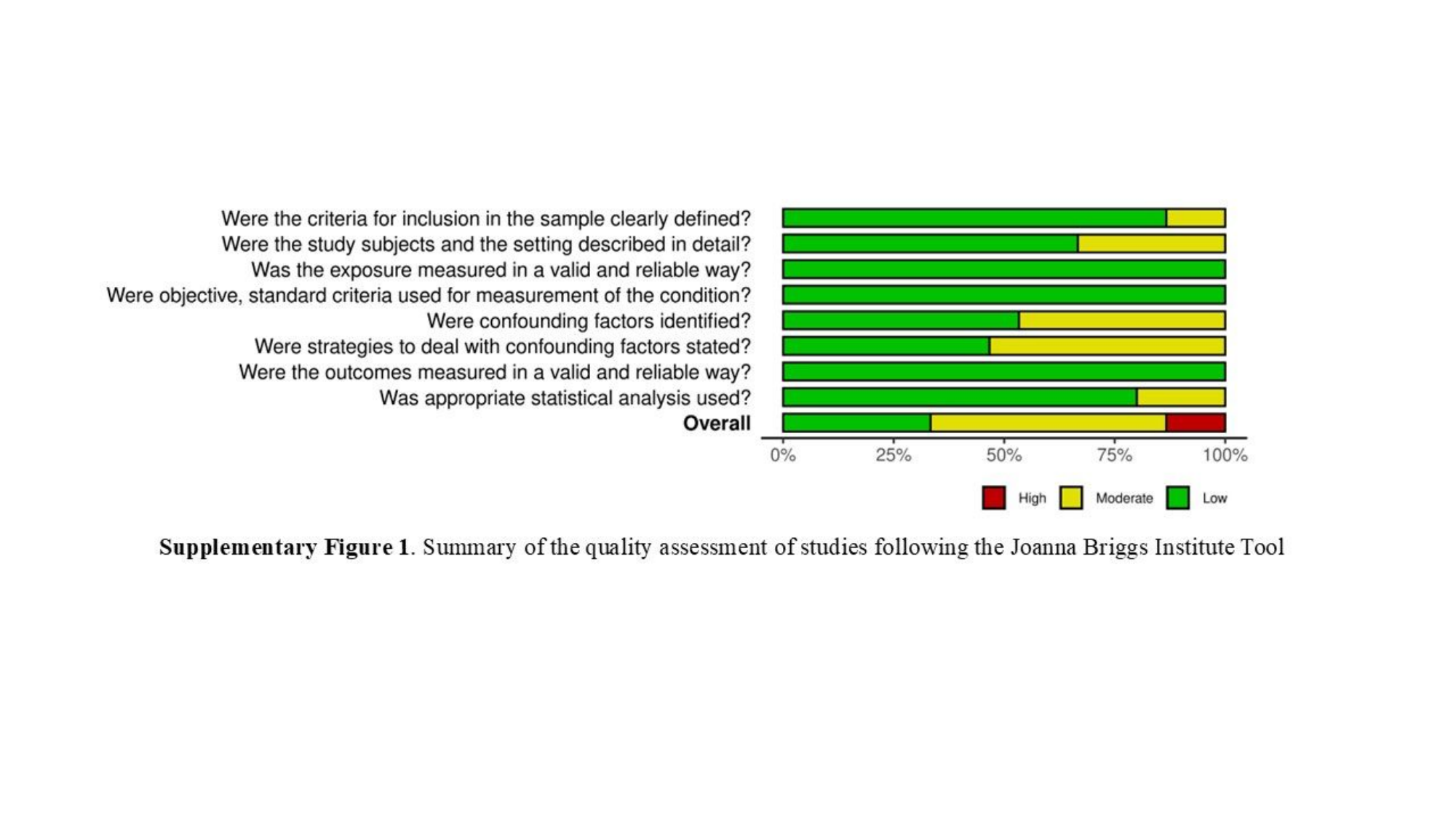

## Slide 2
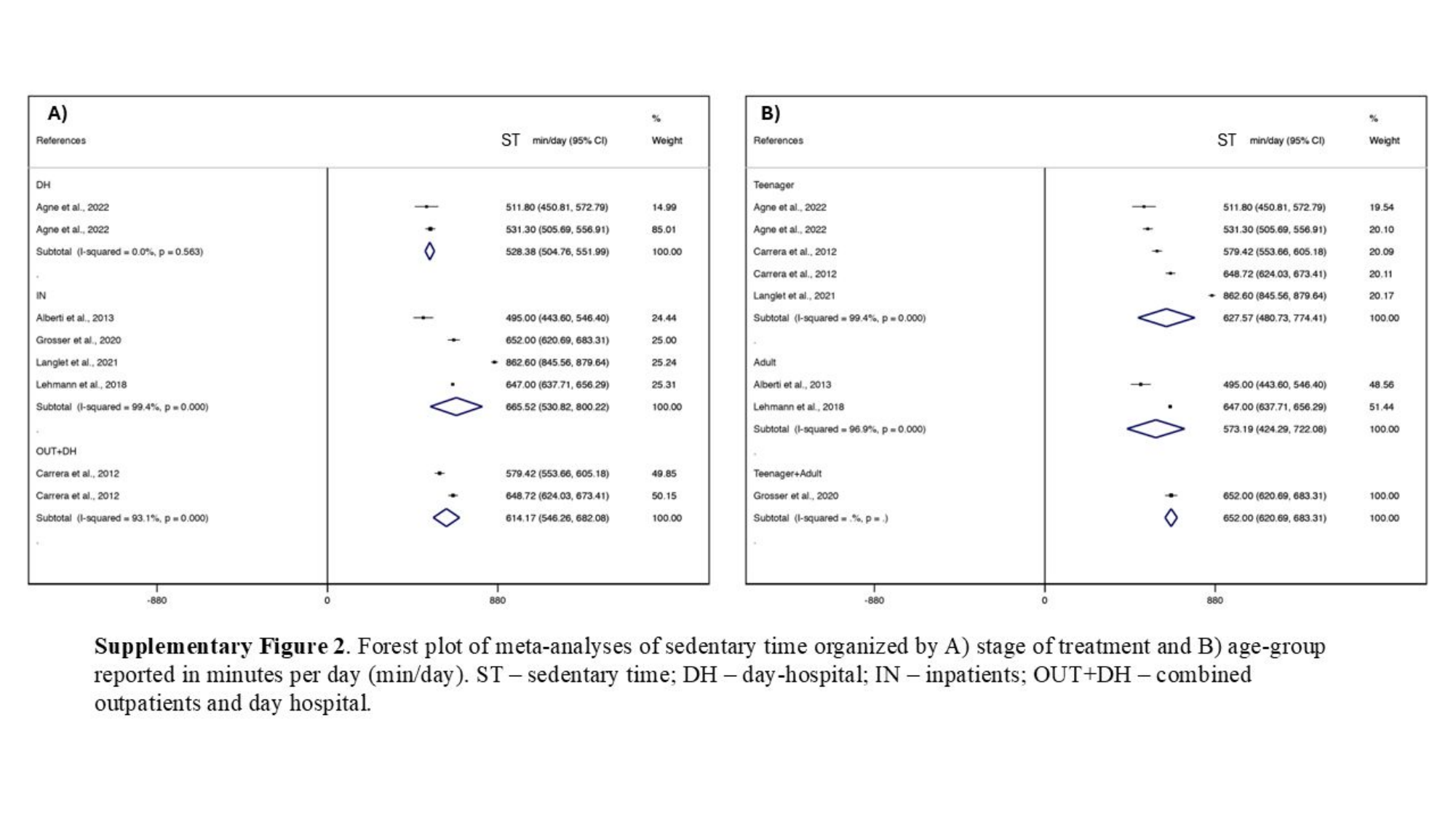

## Slide 3
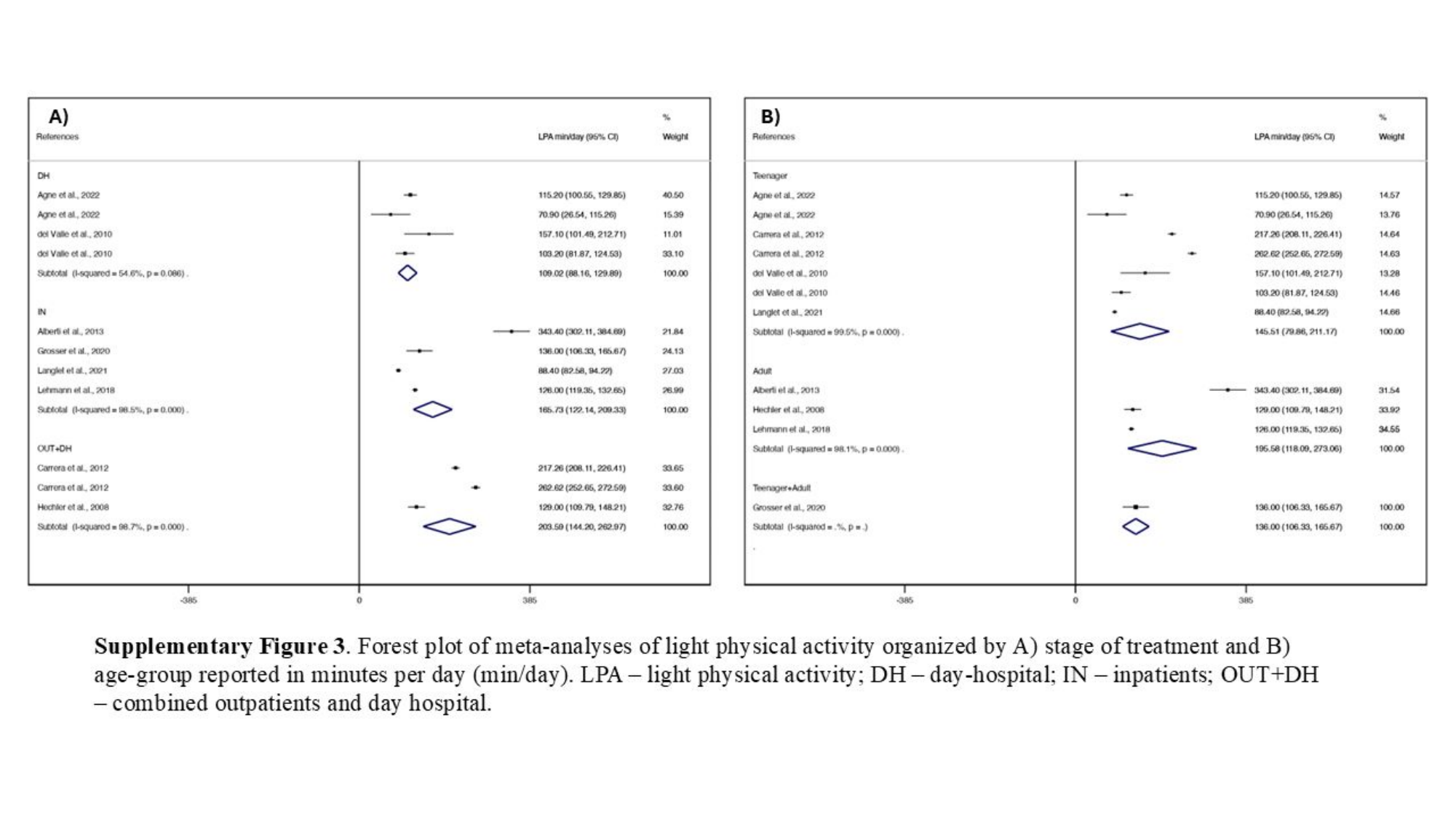

## Slide 4
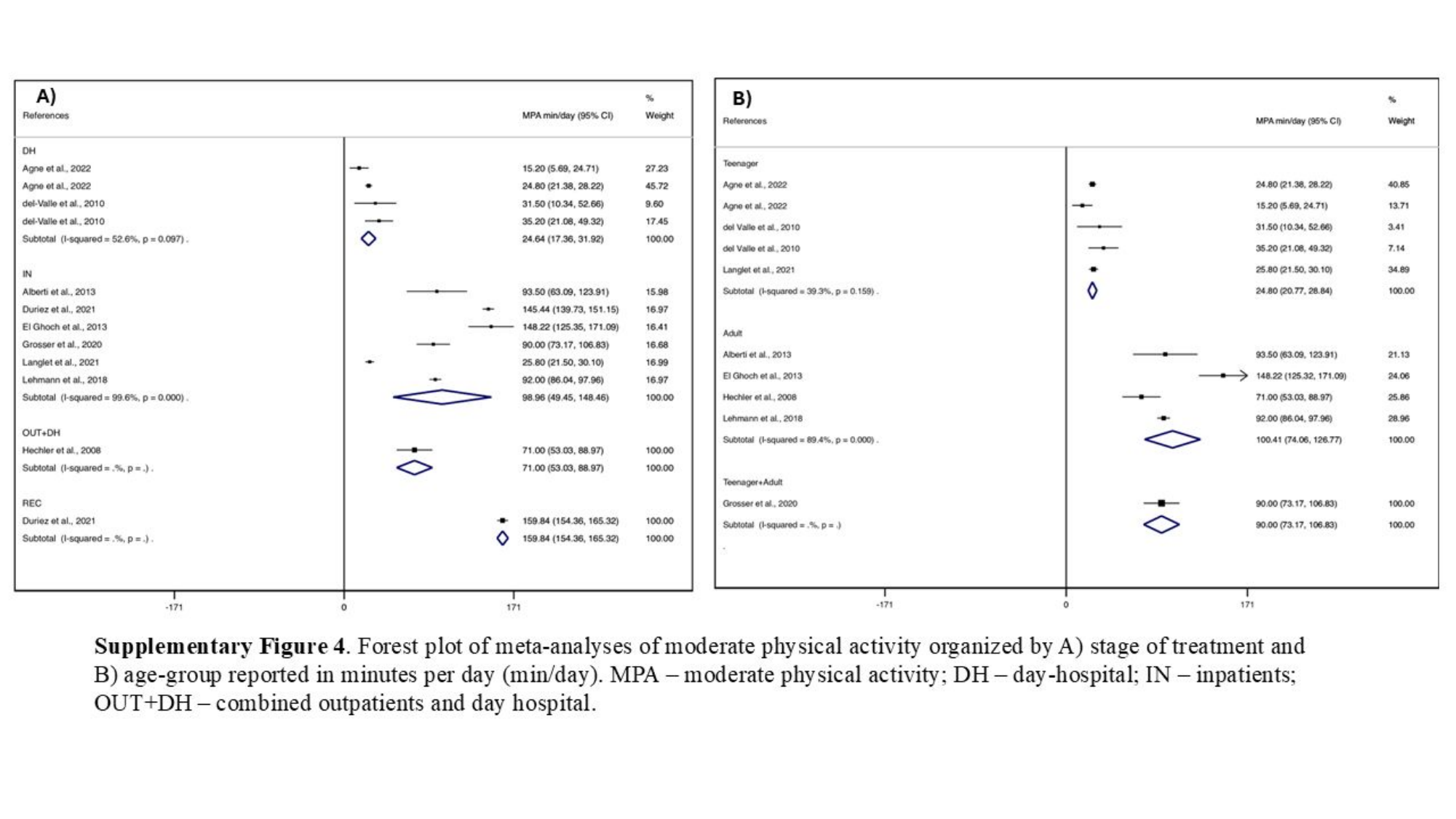

## Slide 5
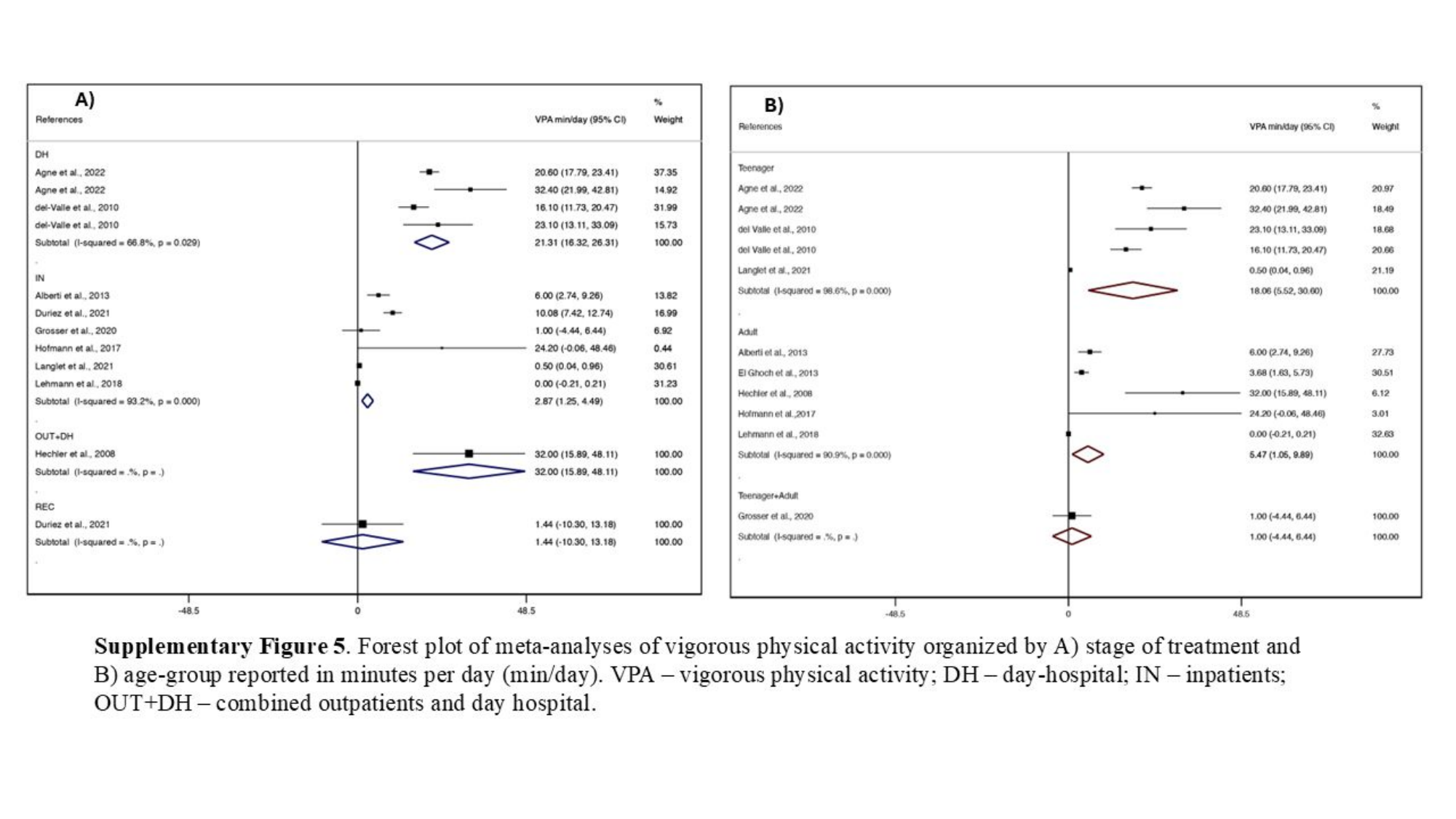

## Slide 6
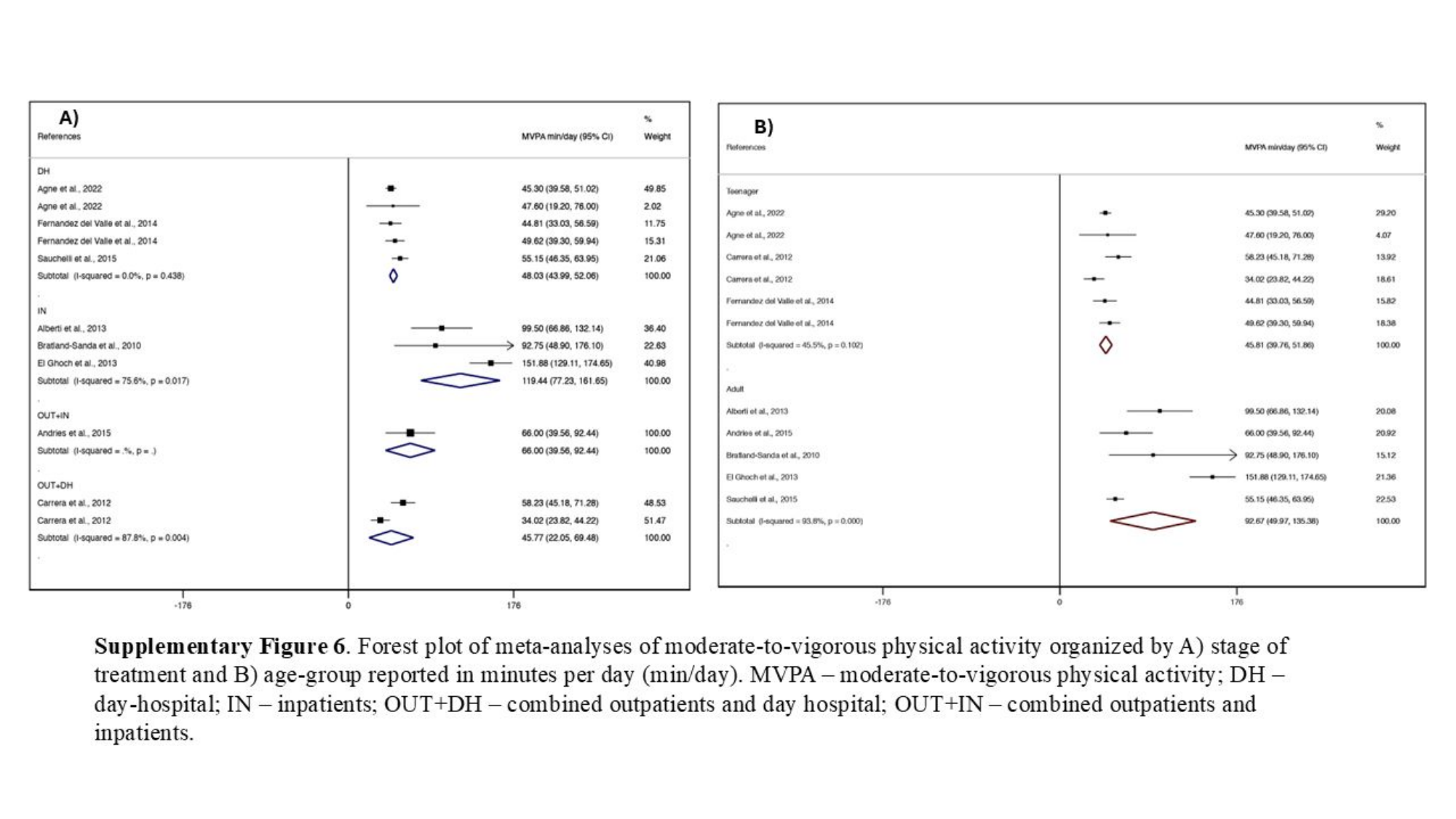

## Slide 7
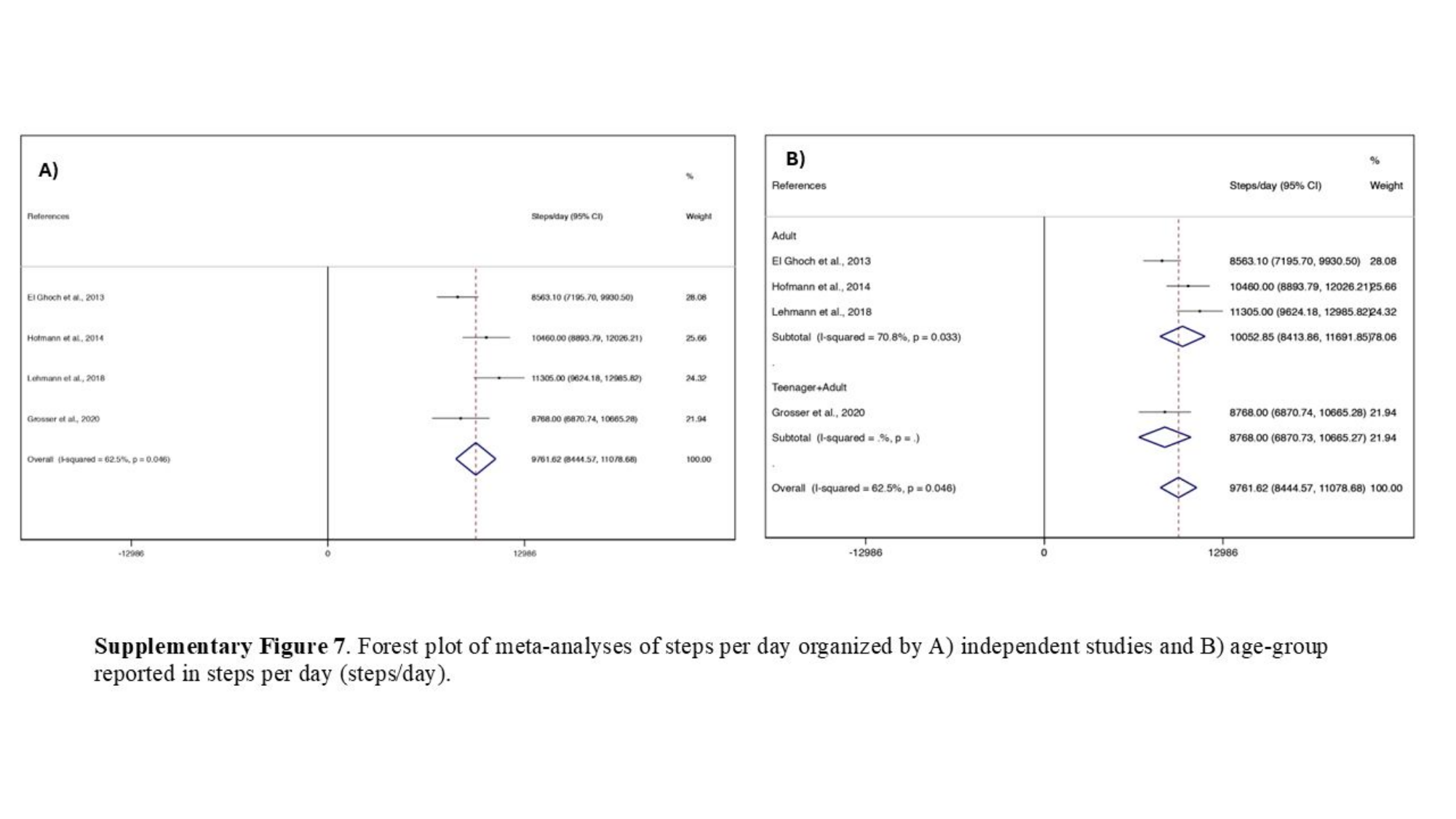

## Slide 8
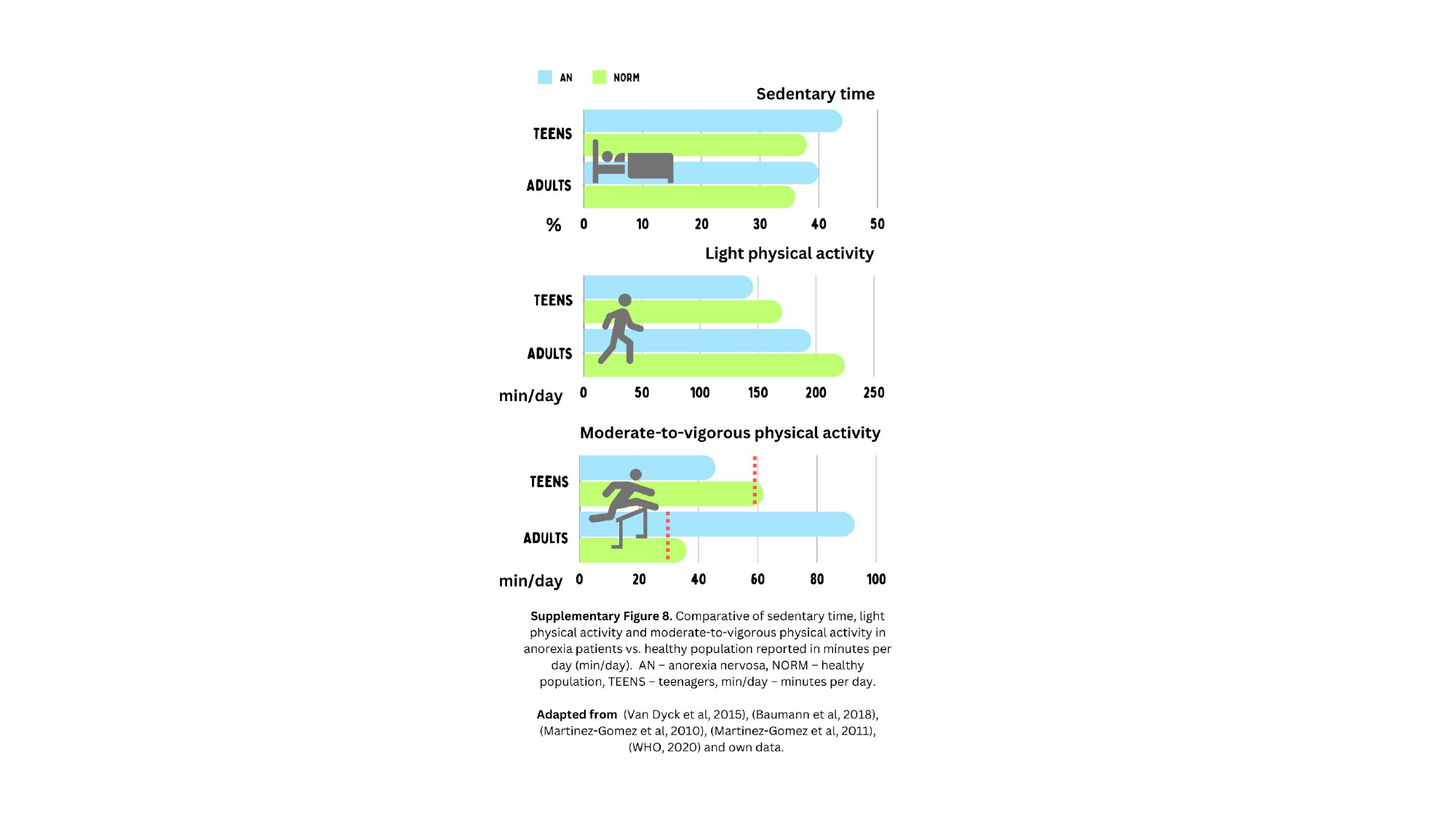

## Slide 9
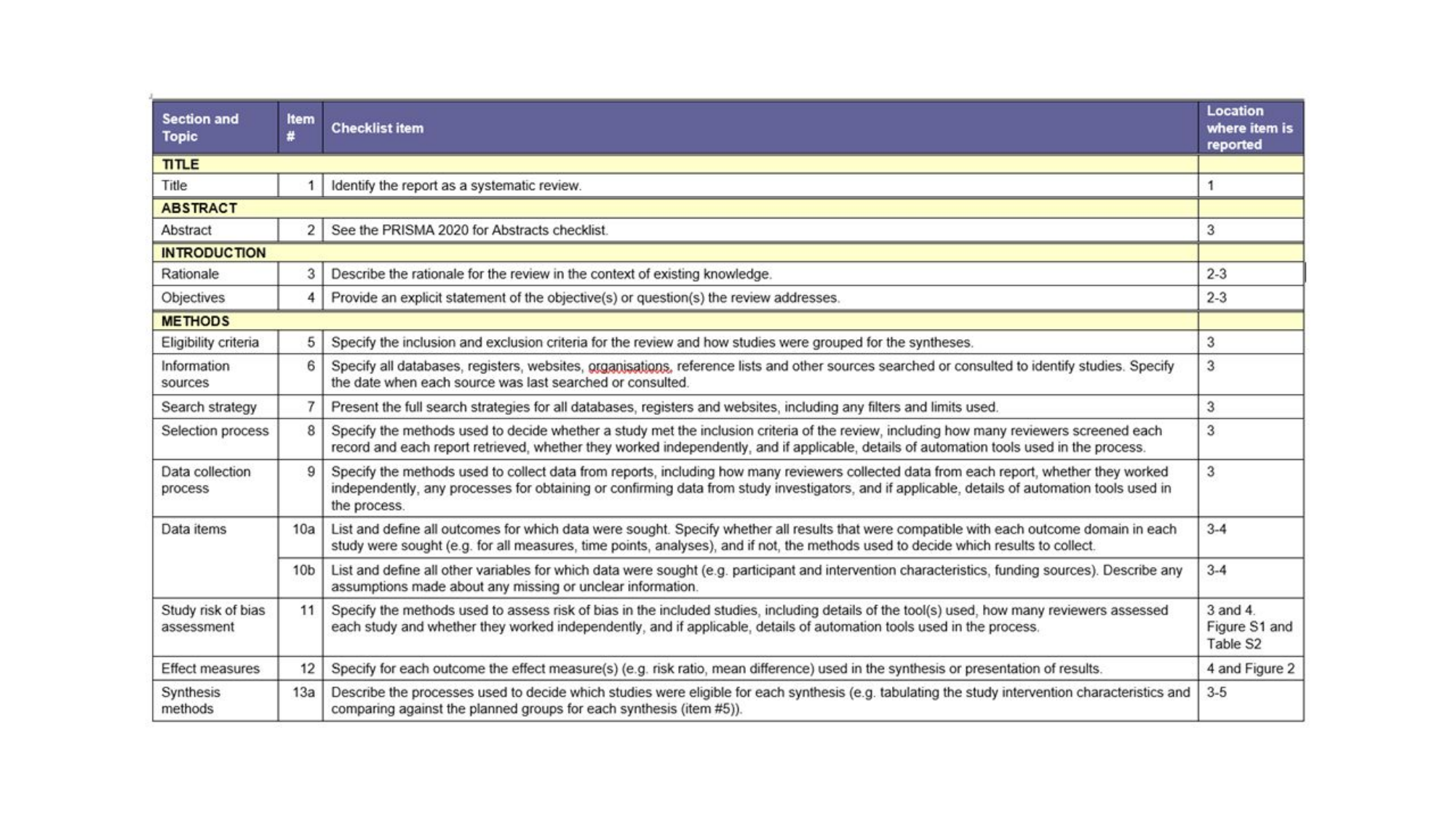

## Slide 10
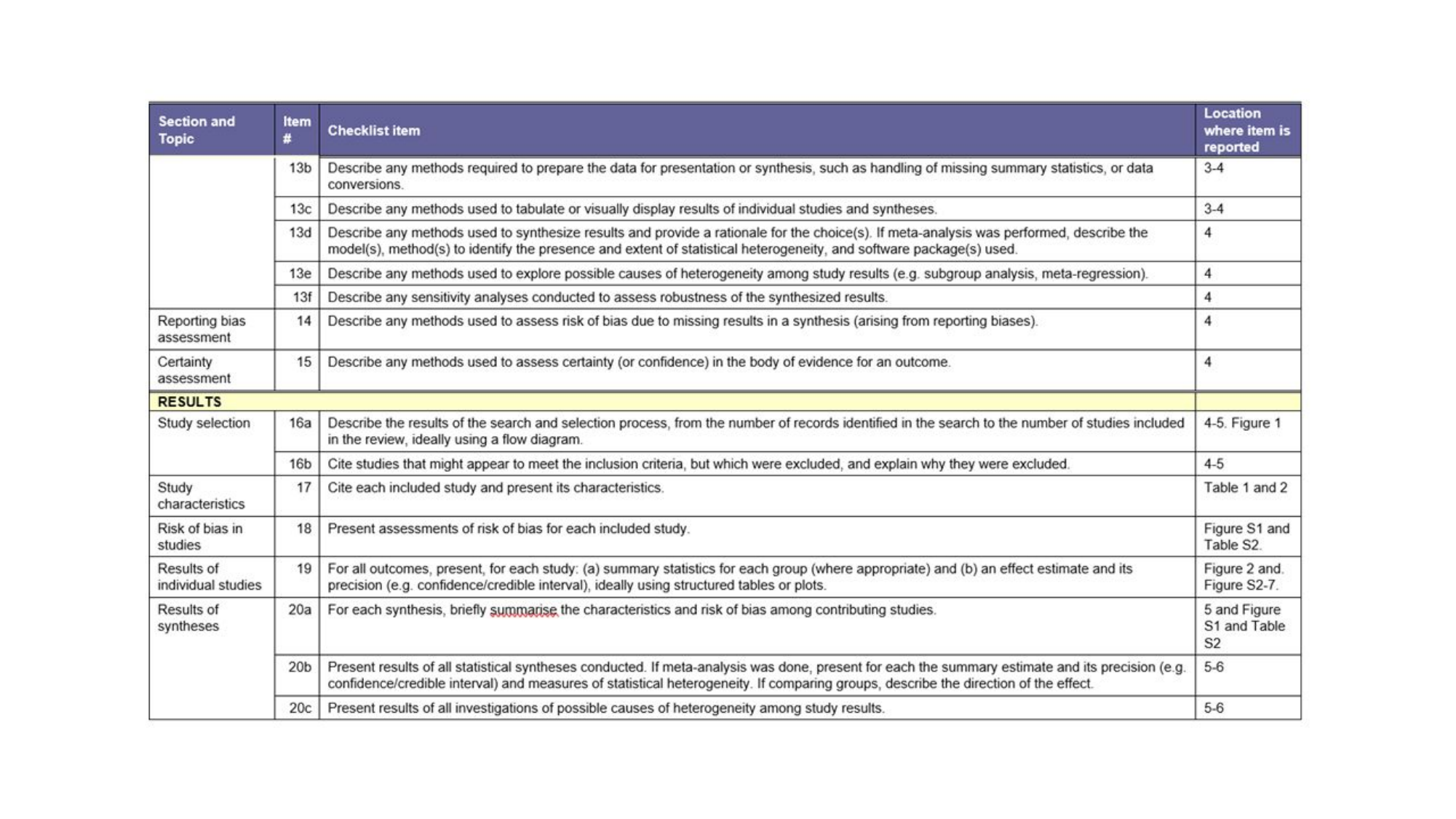

## Slide 11
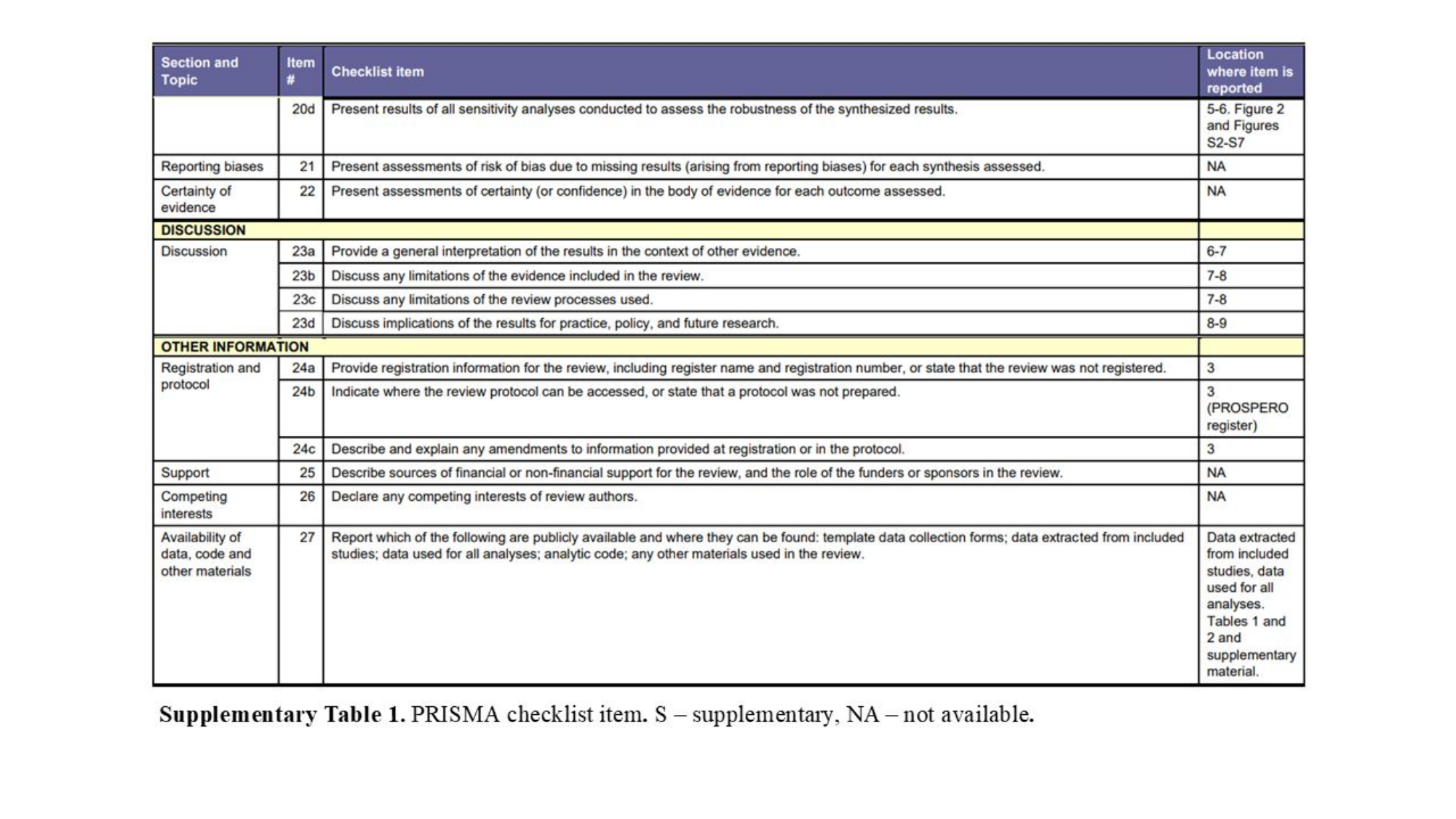

## Slide 12
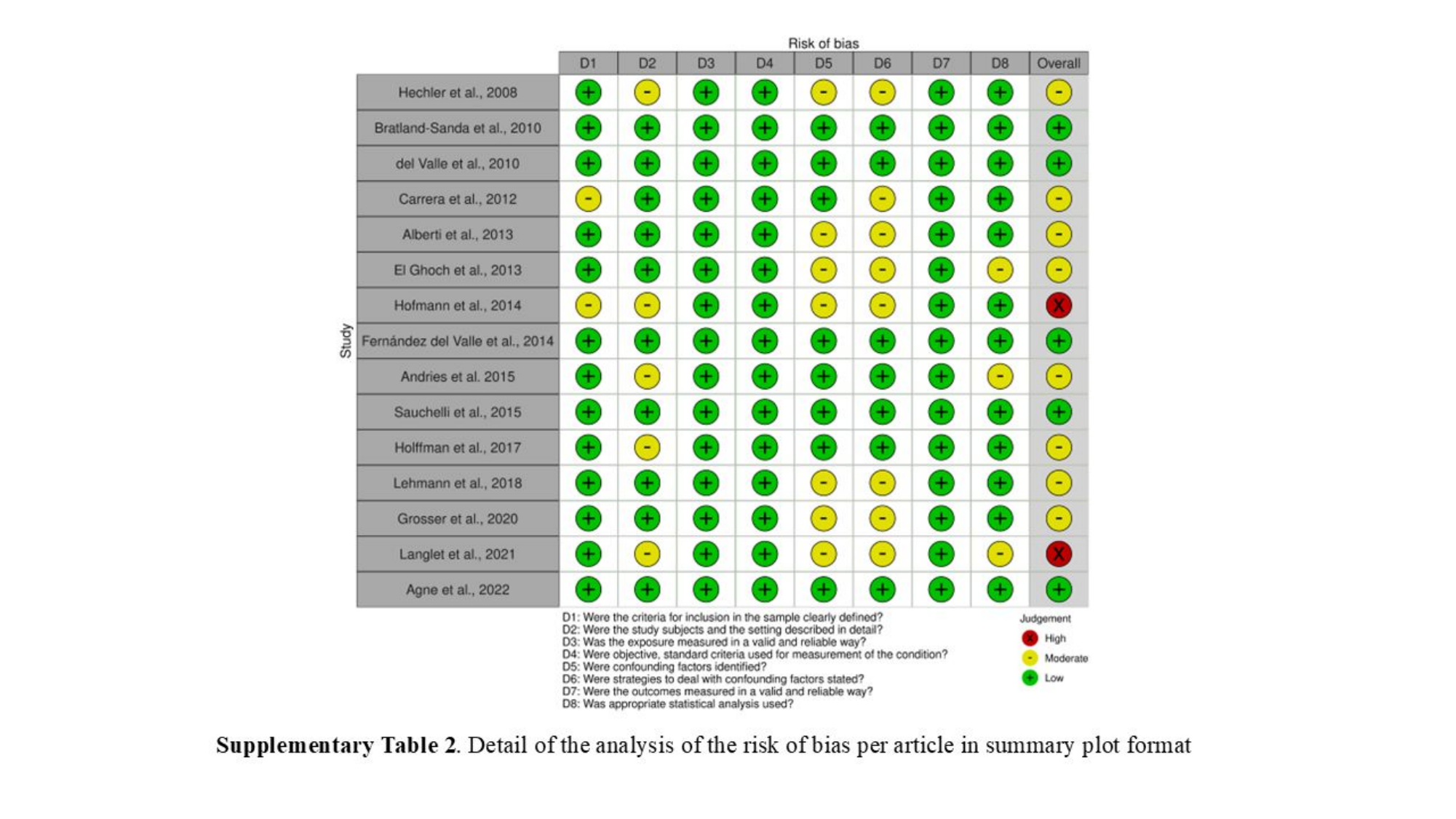

Supplement: Supplementary file 1 — Supporting Information S1 [file ERV-33-1204-s001.pptx]
